# Neurobiological effects of early trauma exposure in people with eating disorders: implications for treatment

**DOI:** 10.1192/j.eurpsy.2023.50

**Published:** 2023-07-19

**Authors:** G. Cascino

**Affiliations:** Medicine, Surgery and Dentistry ‘Scuola Medica Salernitana’, University of Salerno, Baronissi, Italy

## Abstract

**Abstract:**

People with eating disorders (EDs) exhibit a prevalence of childhood maltreatment higher than general population and, as for other psychiatric conditions, a history of childhood maltreatment in the context of EDs has been found associated with an earlier age at onset, a greater clinical severity, a more frequent comorbidity with other psychiatric conditions and a poorer treatment response . Neuroendocrine modifications as well as a heightened biological and emotional vulnerability to acute social stressor exposure and cortical measures alterations have been reported in people with EDs and history of childhood maltreatment. This evidence suggests the possibility to identify a “maltreated ecophenotype” also in people affected by EDs which recommends grouping individuals affected by the same psychiatric condition into subgroups characterized by different clinical and biological correlates in order to tailor treatements.

**Disclosure of Interest:**

None Declared

